# Telemedicine use among physicians in the German outpatient sector: A secondary analysis of a cardiologist-dominated web-based survey with Bayesian model averaging and exploratory machine learning

**DOI:** 10.1177/20552076261460253

**Published:** 2026-07-16

**Authors:** Pascal Petit, Nicolas Vuillerme, Jan Gehrmann, Johannes Stephan, Felix Mühlensiepen, Jonathan Nübel, Eimo Martens, Franziska Hahn

**Affiliations:** 1 Univ. Grenoble Alpes, CNRS, Grenoble INP, LIG, SANGRIA, Grenoble, France; 2 Institut Universitaire de France, Paris, France; 3 TUM School of Medicine and Health, Social Determinants of Health, 9184 Technical University of Munich, Munich, Germany; 4 TUM School of Medicine and Health, Institute of General Practice and Health Services Research, 9184 Technical University of Munich, Munich, Germany; 5 Center for Health Services Research, Faculty of Health Sciences Brandenburg, Brandenburg Medical School, Rüdersdorf bei Berlin, Brandenburg, Germany; 6 Corporate Member of Freie Universität Berlin and Humboldt-Universität zu Berlin, 14903 Charité – Universitätsmedizin Berlin, Berlin, Germany; 7 Department of Intensive Care, 96043 Austin Hospital, Heidelberg, VIC, Australia; 8 TUM School of Medicine and Health, Department Clinical Medicine, Department of Internal Medicine I, 9184 Technical University of Munich, Munich, Germany; 9 European Reference Network Guard Heart, Amsterdam, Netherlands; 10 German Telemedicine Society (DG Telemed), Berlin, Germany

**Keywords:** e-health, telemedicine, outpatient care, digital health, cardiology, machine learning, data reuse

## Abstract

**Background:**

Cardiovascular diseases remain a major health burden in Germany, and telemedicine (TM) offers promising solutions for outpatient care, yet barriers limit uptake. While prior studies relied on qualitative or conventional statistical methods, they often struggled with model uncertainty and complex relationships. Building on a national survey, this study applies Bayesian Model Averaging (BMA) and extreme gradient boosting (XGBoost).

**Objectives:**

This study aimed to explore candidate associations and patterns related to TM use among physicians in the German outpatient sector.

**Methods:**

We conducted a secondary analysis of a web-based survey carried out between 2023 and 2024. BMA was applied to identify explanatory associations with TM use, explicitly accounting for model uncertainty. XGBoost with SHAP values was used to explore classification patterns in a hypothesis-generating framework using repeated nested cross-validation.

**Results:**

Of the 165 respondents, 95 (58%) reported using TM. BMA revealed a limited number of variables with moderate to high posterior inclusion probabilities (PIP), alongside substantial overall model uncertainty, with TM use associated with receiving information from professional associations or insurers and perceived TM benefits (e.g., improving patients’ everyday quality of life, improving doctor-patient relationship). Practicing in Lower Saxony was associated with lower TM use. XGBoost demonstrated limited discriminative ability, with performance statistically indistinguishable from chance. SHAP-based analyses therefore identified exploratory patterns, including features such as information status, workplace, perceptions of TM’s benefits for patients (e.g., health literacy, compliance and adherence) and TM’s barriers (e.g., data protection, implementation incompatibility), as well as employment status.

**Conclusion:**

TM adoption in Germany’s outpatient sector appears associated with structural–economic factors and physicians’ perceptions of patient-related benefits. However, given the substantial model uncertainty in BMA and the limited predictive performance of the machine learning model, all findings should be interpreted cautiously. The machine learning component should be considered exploratory and hypothesis-generating.

## 1. Introduction

Cardiovascular diseases remain one of the leading causes of morbidity and mortality in Germany, posing a substantial challenge to the healthcare system.^1,2^ The outpatient sector plays a central role in the long-term management of these patients, yet structural and organizational constraints often affect the continuity and quality of care.^3–7^ In this context, telemedicine (TM) has gained relevance for cardiology, offering solutions such as remote monitoring of heart failure patients, telecardiology consultations, and the integration of wearable devices.^8–10^ TM holds potential to reduce hospitalizations, improve quality of life, and enhance patient safety.^11–14^ At the same time, implementation has raised concerns regarding equity, data protection, and quality of care, highlighting TM’s dual role as both an opportunity and a challenge, particularly during rapid implementation in the COVID-19 pandemic.^15^

International evidence shows that even in highly standardized cardiology settings, the implementation of remote monitoring varies substantially across health systems. In 2025, an international survey on CIED follow-up showed marked differences between the United States and Europe in remote monitoring adoption and follow-up frequency, underscoring that TM uptake is strongly shaped by structural and reimbursement-related factors.^16^ In Germany, systematic implementation in routine outpatient practice remains uneven and is hindered by barriers including technical infrastructure, data protection concerns, reimbursement structures, and varying levels of professional acceptance.^17–21^ In 2020, the German Federal Joint Committee (G-BA) issued a landmark resolution on the telemedical care of patients, establishing the legal framework for integration into routine outpatient care.^22^ Instead of a stand-alone telemedical center (TMZ) model, the G-BA envisions a network-based structure in which telemedical functions are embedded within existing outpatient workflows. This network comprises TMZs and Primary Treating Physicians (PBAs), typically involving office-based cardiologists and their associated PBAs, who may also be internists, general practitioners, or nephrologists. In most cases, cardiologists assume both roles, reflecting the intention to integrate telemedical monitoring into established care pathways rather than promote independent TMZs. This highlights the need to identify factors influencing TM use under these structural conditions.

A variety of approaches have been employed to better understand factors associated with TM adoption among healthcare professionals (HCPs). Most studies relied on qualitative methods,^23–26^ descriptive statistics,^27,28^ clustering techniques,^18,29^ or logistic regression models.^30–32^ While these approaches have generated valuable insights, they also face important limitations, particularly in handling model uncertainty, achieving stable predictive performance, and capturing complex nonlinear relationships between predictors and outcomes.

To address these limitations, two complementary approaches stand out: Bayesian model averaging (BMA) and modern machine learning (ML) techniques, particularly tree-based algorithms. BMA is well suited for identifying candidate variables statistically associated with an outcome such as TM use, while accounting for model uncertainty. In contrast, ML methods such as extreme gradient boosting (XGBoost) are primarily designed to optimize predictive accuracy and can uncover complex, nonlinear, and high-dimensional relationships that are often overlooked by conventional regression approaches. Unlike traditional regression analyses, which typically select a single “best-fitting” model and disregard the uncertainty inherent in model specification, BMA formally incorporates model uncertainty by averaging across a set of plausible models weighted by their posterior probabilities.^33,34^ XGBoost, for its part, is widely recognized for its predictive accuracy, computational efficiency, and the ability to model nonlinear dependencies.^35–37^ However, the performance of ML models is strongly dependent on sample size and signal strength. In relatively small datasets with a high number of features, predictive models may exhibit limited discrimination and high variability, which constrains their interpretability and practical utility.

The present study builds on a previously published survey among cardiologists, internists, and general practitioners in Germany, which examined the current use, acceptance, opportunities, and barriers related to TM in the outpatient sector.^29^ That initial analysis revealed a high level of acceptance of TM, but also highlighted major challenges, particularly regarding technical infrastructure, reimbursement, and information gaps. It further identified four distinct user clusters (‘pioneers,’ ‘focused practitioners,’ ‘using skeptics,’ and ‘uninformed distanced’), each characterized by specific attitudes, needs, and barriers toward TM. The aim of this secondary analysis is to move beyond descriptive findings and apply advanced statistical and ML methods to identify candidate associations and exploratory predictors of actual TM adoption in the outpatient sector to empower HCPs and stakeholders. We adopt a data-driven strategy, not guided by a single a priori technology adoption framework, to disentangle explanatory relevance from predictive relevance. To this end, we combine BMA and XGBoost to generate explanatory and exploratory predictive insights while acknowledging the constraints imposed by sample size and model performance.

## 2. Materials & methods

### 2.1. Study design

The dataset used for this secondary analysis was derived from a previously published web-based survey by Gehrmann et al. (2025), which investigated the needs, attitudes, barriers, and facilitators of TM use among cardiologists, internists, and general practitioners in Germany.^29^ A 32-item questionnaire covering current use, opportunities, challenges, and needs was distributed via professional associations, including the German Cardiac Society, the Association of Registered Cardiologists, and the German Society of General Practice and Family Medicine. Due to this distribution strategy, the total number of physicians who received the survey invitation could not be determined, and a response rate cannot be calculated. The involved associations together represent a membership base of approximately 20,000 physicians across the involved professional associations.^38–40^ Data were collected from October 3, 2023, to January 31, 2024. Ethical approval for the study was obtained from the Ethics Committee of the Medical Faculty of TUM (Ref. 2023-437-S-SB; Date: August 7, 2023). No formal prospective power analysis was conducted for this secondary analysis.

### 2.2. Inclusion criteria

Eligible participants were physicians specialized in cardiology or internal medicine, as well as general practitioners working in the outpatient sector in Germany. Additional eligibility requirements included sufficient proficiency in the German language and the provision of informed consent, obtained prior to study participation via the online survey platform.

### 2.3. Statistical analysis

All analyses were conducted in R (version 4.5.2®) on Windows 11©. TM use (yes/no) served as the outcome variable. The analysis was data-driven and not based on a single pre-specified technology adoption framework.

#### 2.3.1. Data management

Because the dataset did not include a binary variable for TM use, we created one. Participants were classified as TM users if they reported using at least one TM technology (e.g., video consultations, wearables), corresponding to at least one “yes” response to questions A102_01 through A102_05. These items captured the use of video consultations (A102_01), monitoring applications enabling continuous and location-independent tracking of vital parameters (A102_02), health-related mobile applications (A102_03), wearable devices (e.g., smartwatches) (A102_04), and other telemedical applications (A102_05).

Independent variables were defined based on all variables except TM use. Ordered variables were retained as ordered factors without contrast coding. All nominal variables were one-hot encoded, with one category removed per variable to avoid collinearity.^41^ Missing data were imputed using the MissForest algorithm, a single-imputation method capable of handling both categorical and continuous variables while capturing nonlinear relationships by leveraging the ensemble structure of random forests to estimate imputation error via out of bag estimates.^42^ Before imputation, variables with more than 30% missing data were excluded to enhance dataset robustness and minimize bias due to excessive missingness. On average, 7.56% of data were missing per predictor (95% CI: 0–24.7). To further reduce missingness, records with more than 50% missing data (7 out of 172) and one variable (out of 77) with more than 30% missing data were discarded.

#### 2.3.2. BMA analysis

To identify candidate associations with TM use, we applied a structured three-step analytical approach consisting of (1) collinearity screening, (2) variable selection using Least Absolute Shrinkage and Selection Operator (LASSO) regression, and (3) BMA.

To reduce dimensionality and ensure the reliability of coefficient estimates, we first conducted collinearity screening. Pairwise correlations were examined, and the variance inflation factor (VIF) was calculated for all candidate variables. Variables with a VIF greater than 5 were excluded to mitigate multicollinearity. The resulting set of non-collinear variables was then subjected to LASSO regression, which applies an L1 penalty to shrink less relevant coefficients toward zero, retaining only the most informative variables. Five-fold cross-validation (CV) was used to identify the optimal penalization parameter (lambda), based on the minimization of cross-validated binomial deviance. Variables with non-zero coefficients at the optimal lambda were retained for further analysis. The rationale for applying LASSO prior to BMA was pragmatic: given the relatively small sample size (n = 165) and large number of candidate variables (n = 77), this step reduced dimensionality, limited model space explosion, and helped mitigate signal dilution. However, restricting the set of candidate variables before BMA means that posterior inclusion probabilities (PIPs) reflect conditional uncertainty within a pre-selected subset rather than uncertainty across the full model space. The selected variables were then entered into a BMA framework using logistic regression. BMA addresses model uncertainty by averaging across all possible models, weighted by their posterior probabilities, rather than relying on a single best-fitting model.^43^ This approach yields PIPs for each variable, representing the probability that the corresponding covariate has a non-zero effect on TM use. PIPs were treated as a continuous measure of evidence, with higher values indicating stronger evidence. Given the limited sample size and reduced model space, PIPs should be interpreted cautiously, particularly for variables with low values, which reflect weak and potentially unstable evidence. BMA was performed using the BMA package,^44^ with equal prior probability assigned to all candidate models.

#### 2.3.3. XGBoost analysis

To complement the BMA analysis and leverage the exploratory capacity of ML, we applied an XGBoost binary classification model. Unlike the BMA pipeline, no collinearity filtering was performed prior to model fitting, as tree-based algorithms are inherently robust to multicollinearity through hierarchical partitioning and built-in regularization mechanisms.^35–37^ Given the relatively small sample size and high-dimensional feature space, the ML analysis was explicitly designed as hypothesis-generating rather than confirmatory.

To estimate predictive performance under repeated nested CV, we performed 50 repeats of 5-fold outer CV (stratified sampling). Within each outer training fold, hyperparameter tuning used 3-fold inner CV across 30 random hyperparameter combinations sampled from predefined ranges: learning_rate (0.01-0.3), subsample (0.1-1.0), colsample_bytree (0.5-1.0), min_child_weight (1-10), max_depth (1-10), and scale_pos_weight, which was dynamically set to the ratio of majority to minority class to account for class imbalance.

Hyperparameter selection was optimized based on maximizing the mean area under the receiver operating characteristic curve (AUROC) across inner folds. The optimal hyperparameter configuration identified within the inner loop was then used to train a model on the full outer training fold, which was subsequently evaluated on the corresponding outer test fold. This process was repeated across all folds and repetitions, yielding a distribution of performance metrics across 250 outer test folds. Performance was summarized using AUROC, accuracy, sensitivity, specificity, and the F1 score. Each metric was summarized using mean values with empirical 95% confidence intervals derived from the repeated CV results. Model calibration was additionally assessed using the Brier score. Given the sample size and resulting fold structure, performance estimates are subject to substantial variability and should be interpreted as approximate rather than definitive measures of predictive ability.

The XGBoost algorithm was implemented using the xgboost package.^45^ Model training, CV, and hyperparameter tuning were carried out with the mlexperiments package^46^ in combination with the mllrnrs package,^47^ which provide standardized ML pipelines and learner interfaces. Data partitioning and fold creation were performed using the splitTools package.^48^ Model performance was evaluated with the pROC package^49^ for AUROC estimation and MLmetrics package.^50^

#### 2.3.4. SHAP analysis

To enhance interpretability of the XGBoost model, SHapley Additive exPlanations (SHAP) values were computed across all outer test folds and repetitions to quantify the contribution of each predictor to the model output.^51^ For each trained model, SHAP values were calculated on the corresponding outer test set and then aggregated across all folds and repeats to obtain distribution-based estimates of feature importance. For each predictor, mean absolute SHAP values and empirical 95% confidence intervals were computed. Predictors that increased the probability of TM use were considered as potential promoting factors, whereas those that reduced it were considered as potential limiting or dissuasive factors. Directionality was inferred using a hybrid heuristic. For binary features, mean SHAP values were compared across groups, while for continuous features, generalized additive models (GAMs) were fitted to SHAP values, and the average derivative of the fitted curve was used to determine association direction. When GAMs failed to converge, Spearman’s rank correlation between the feature and its SHAP values served as a fallback. This approach is pragmatic but non-standard and introduces additional uncertainty. SHAP-based interpretations were therefore treated as descriptive and hypothesis-generating rather than confirmatory or causal. Conventionally, SHAP directionality is inferred from the sign of the mean SHAP value across all observations for a given feature, or assessed visually through SHAP dependence plots. However, both approaches can be misleading or difficult to interpret for non-monotonic features or those exhibiting U-shaped or threshold relationships. We therefore adopted the heuristic approach previously described, which captures the average directional tendency across the full support of each feature while accommodating non-linearity. For comparison, SHAP directions derived from the sign of the mean SHAP value are also reported.

## 3. Results

### 3.1. Participant characteristics

Of 165 respondents, 95 (59%) reported using TM. Participants included cardiologists (77.0%), internists (15.2%), and general practitioners (7.9%). Most were male (75.2%) and over 40 years old (40–49: 21.2%, 50–59: 42.4%, >60: 29.1%). State-level sample sizes are reported in Table 1 to allow assessment of the robustness of regional effects. Several federal states were represented by relatively small numbers of participants, which should be taken into account when interpreting geographic associations. For detailed demographics, see Gehrmann et al. 2025.^29^

**Table 1.** State-level number of practitioners included.

State	n	%
Bavaria	50	30.3
Lower Saxony	30	18.2
Baden-Württemberg	18	10.9
Mecklenburg-Western Pomerania	15	9.09
Saarland	9	5.45
Hamburg	9	5.45
Berlin	7	4.24
Hesse	6	3.64
North Rhine-Westphalia	5	3.03
Bremen	5	3.03
Saxony	3	1.82
Schleswig-Holstein	3	1.82
Saxony-Anhalt	2	1.21
Brandenburg	2	1.21
Rhineland-Palatinate	1	0.61

### 3.2. BMA analysis

Of the initial 77 covariates, 56 were retained after collinearity filtering based on VIF and 12 variables were subsequently selected through LASSO regression for inclusion in the BMA analysis (Table 2).

**Table 2.** TM use candidate associations identified through BMA.

Candidate association	Top model	Top 5 models	BMA		Posterior inclusion Probability (%)	Direction
	Mean	Mean	Mean	sd		
Federal State in Germany - Lower Saxony	-0.86	-1.00	-0.86	0.61	76.9	Negative association
Acceptance: Telemedicine improves patients’ everyday quality of life	0.32	0.50	0.32	0.33	56.7	Positive association
Acceptance: Telemedicine improves the doctor-patient relationship	0.26	0.50	0.26	0.30	50.6	Positive association
Information requirements: Professional associations	0.00	0.36	0.27	0.41	37.5	Positive association
Information requirements: Health insurance companies	0.00	0.00	0.15	0.36	19.4	Positive association
Information sources: Professional associations	0.00	0.15	0.13	0.30	19.1	Positive association
Opportunities and Barriers: Connecting to relevant clinics, centers or service providers involves too much effort	0.00	0.00	-0.06	0.14	18.9	Negative association
Barriers: Acquisition and implementation of telemedicine applications is associated with excessive costs	0.00	0.00	0.06	0.20	10.5	Positive association
Information requirements: Scientific journals	0.00	0.00	0.07	0.24	10.4	Positive association
Information status	0.00	0.00	0.02	0.09	9.00	Positive association
Employment status - employment outside MVZ	0.00	0.00	-0.07	0.31	6.80	Negative association
Opportunities and Barriers: The inadequate reimbursement of telemedicine services is a problem	0.00	0.00	-0.01	0.07	4.30	Negative association

*Note.* MVZ: « Medizinisches Versorgungszentrum » (Medical Care Center); a German outpatient group practice model.

**Figure 1.**
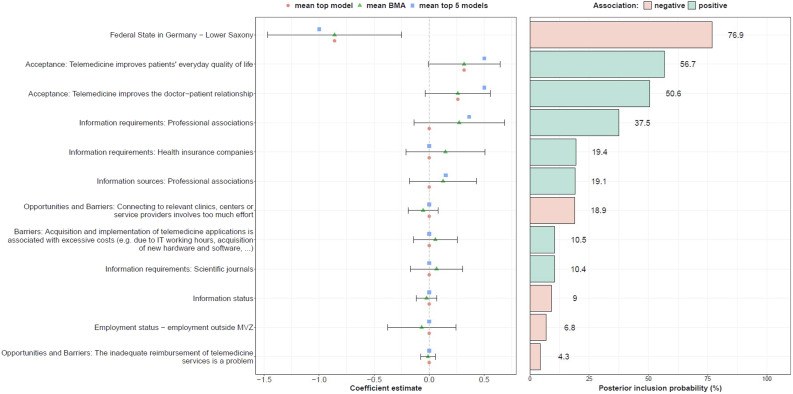
Coefficient estimates for each predictor variable under three modeling perspectives: the highest posterior probability single model (“Top model”), an approximation averaged over the top five models (“Top 5 models”), and the fully Bayesian model-averaged estimates (“BMA”). The points represent the estimated coefficient effect sizes, while the error bars show the posterior standard deviations derived from the full BMA analysis, reflecting uncertainty in these averaged estimates. The “Top model” and “Top 5 models” estimates do not include uncertainty intervals because these approximations do not capture model-averaged uncertainty. The full “BMA” estimates incorporate all plausible models weighted by their posterior probabilities, shrinking coefficients toward zero when evidence for inclusion is weak and properly reflecting parameter uncertainty.

Posterior model probabilities indicated substantial model uncertainty. No single model dominated, with the highest posterior model accounting for only 5.8% of the total weight, and the top five models together representing 18.4%. Estimates based on only the top model, or approximated across the top five models, differed from the fully model-averaged estimates (Table 2, Figure 1). In contrast, posterior means from the full BMA, which incorporate information from the entire model space, provided more stable estimates that were often shrunk toward zero.

Across the 12 variables included in the BMA model, PIPs varied widely. Three variables had PIPs below 10%, five ranged between 10% and 20%, one between 20% and 50%, and three exceeded 50% (Table 2, Figure 1). PIPs were interpreted as continuous measures of evidence, with values below 50% indicating weak to moderate evidence that should be interpreted cautiously.

Among the variables with the strongest evidence, a negative association with TM use was observed for practitioners working in Lower Saxony (PIP = 76.9%). Positive associations included perceiving TM as improving patients’ everyday quality of life (PIP = 56.7%) and improving the doctor-patient relationship (PIP = 50.6%). Additional variables, such as information requirements from professional associations (PIP = 37.5%) and health insurance companies (PIP = 19.4%), showed weaker but non-negligible evidence of association.

### 3.3. XGBoost analysis

The XGBoost model showed limited predictive performance. Across the repeated nested CV procedure, the mean AUROC was 0.53 (95% CI: 0.48-0.61; SD = 0.03), indicating discrimination only marginally above chance. The confidence interval crossed 0.50, indicating that the model did not reliably distinguish between TM users and non-users. Additional performance metrics were as follows: accuracy = 0.53 (95% CI: 0.45-0.60; SD = 0.04), sensitivity = 41.9% (95% CI: 27.4-57.7%; SD = 0.08), specificity = 57.3% (95% CI: 52.5-62.7%; SD = 0.03), and F1 score = 0.32 (95% CI: 0.10-0.47; SD = 0.10). Model calibration was limited, with a Brier score of 0.25. Overall, the model provided limited predictive signal and should be interpreted as hypothesis-generating rather than suitable for decision-making.

A total of 53 features were included in the XGBoost model. Several variables identified in the BMA analysis also appeared among the more important SHAP features, but this apparent convergence should be interpreted cautiously given the limited model performance. In addition to overlapping variables, the SHAP analysis identified several additional features that did not emerge in the BMA framework (Table 3), including perceived barriers to TM use (e.g., data protection concerns, need for training, implementation incompatibility, perceived effort, and risk of misdiagnosis), perceived benefits (e.g., improvements in patient health literacy, compliance, and adherence), and workplace. These findings likely reflect the ability of tree-based models to capture complex, nonlinear, and interaction effects that are not explicitly modeled in regression-based approaches.

**Table 3.** Top 20 features with the highest mean absolute SHAP values.

Feature	Mean |SHAP| (95% CI)	Direction (heuristic)	Direction (conventional)
Information status	0.060 (3.42e-4; 0.305)	limiting	promoting
Information requirements: Professional associations	0.050 (0; 0.340)	promoting	limiting
Information sources: Professional associations	0.041 (0; 0.295)	promoting	promoting
Acceptance: Telemedicine increases patients’ health literacy	0.036 (0; 0.233)	promoting	promoting
Opportunities and Barriers: The topic of data protection hinders the use of telemedicine applications	0.035 (6.57e-6; 0.218)	limiting	limiting
Acceptance: Telemedicine improves patients’ everyday quality of life	0.035 (0; 0.190)	promoting	limiting
Opportunities and Barriers: Connecting to relevant clinics, centers or service providers involves too much effort	0.032 (0; 0.176)	limiting	limiting
Acceptance: Telemedicine improves the doctor-patient relationship	0.031 (0; 0.180)	promoting	promoting
Opportunities and Barriers: The lack of experience and expertise of medical staff hinders the use of telemedicine applications	0.031 (3.04e-5; 0.154)	limiting	promoting
Barriers: Acquisition and implementation of telemedicine applications is associated with excessive costs (e.g. due to IT working hours, acquisition of new hardware and software)	0.030 (0; 0.212)	promoting	promoting
Working place	0.030 (5.10e-4; 0.148)	limiting	promoting
Opportunities: Telemedicine carries the potential risk of misdiagnoses and missed diagnoses	0.028 (0; 0.200)	limiting	limiting
Opportunities and Barriers: The technical infrastructures hinder the use of telemedicine applications	0.028 (0; 0.155)	limiting	limiting
Opportunities: Telemedicine enables better networking regarding data exchange and its use (integration of health apps, wearables, data exchange with other specialists, etc.)	0.028 (0; 0.150)	promoting	promoting
Acceptance: Telemedicine increases patient compliance and adherence	0.027 (0; 0.167)	promoting	promoting
Acceptance: There is a high level of acceptance of telemedicine applications among patients	0.026 (0; 0.163)	promoting	limiting
Opportunities: Telemedicine enables low-threshold contact with medical staff or patients	0.026 (0; 0.127)	promoting	promoting
Opportunities and Barriers: The introduction of new, especially digital, applications is fundamentally difficult in healthcare	0.025 (0; 0.152)	limiting	limiting
Federal State in Germany - Lower Saxony	0.025 (0; 0.193)	limiting	promoting
Opportunities: Telemedicine leads to resource-oriented care of patients	0.025 (3.34e-5; 0.140)	promoting	promoting

Regarding directionality of SHAP, mean comparisons were applied to 30 features, while Spearman correlations were used for 23 features when GAM did not converge. SHAP results suggested that factors such as information from professional associations, perceived improvements in patient-related outcomes, and enhanced doctor-patient interaction may promote TM use. Conversely, factors such as limited information status, workplace, regional context, lack of experience, need for training, and concerns related to data protection, usability, and diagnostic safety may act as limiting factors.

**Figure 2.**
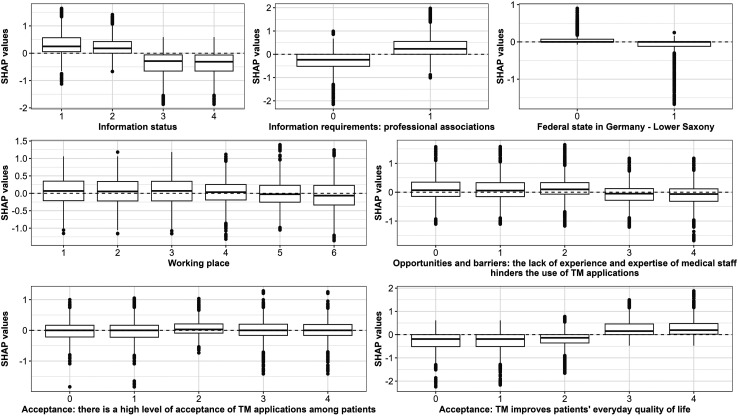
SHAP dependence plots for features among the top 20 by mean absolute SHAP value for which the heuristic and conventional approaches assigned discordant directionality. Note. The x-axis values represent the following response categories: Information status: 1 = Yes, 2 = Rather yes, 3 = Rather no, 4 = No; Information requirements: Professional associations: 0 = Not selected, 1 = Selected; Federal state in Germany – Lower Saxony: 0 = Other federal state, 1 = Lower Saxony; Working place: 1 = <5,000 inhabitants (rural municipality), 2 = 5,000–10,000 (small town), 3 = 10,000–50,000 (small medium-sized town), 4 = 50,000–100,000 (large medium-sized town), 5 = 100,000–500,000 (smaller big city), 6 = >500,000 inhabitants (big city); Acceptance items and Opportunities and Barriers item: 0 = Strongly disagree, 1–3 = intermediate values, 4 = Strongly agree.

When comparing the heuristic and conventional approaches, only 17 of 53 features (32.1%) yielded consistent directional assignments. Among the 20 features with the highest mean absolute SHAP values, directional agreement was observed for 13 features. SHAP dependence plots for features showing discordant directionality between the two approaches are presented in Figure 2. For these features, visual inspection of the dependence plots confirmed that the heuristic approach more accurately captured the true SHAP directionality than the conventional mean SHAP sign method.

## 4. Discussion

### 4.1. Main findings

This study applied two complementary approaches to investigate candidate associations with TM use among physicians in the outpatient sector in Germany, namely BMA as an explanatory framework and XGBoost as a ML-based, hypothesis-generating framework. The two approaches yielded partly different but complementary perspectives. BMA identified a limited number of variables with stronger evidence of association, whereas XGBoost showed limited predictive performance and SHAP provided only exploratory patterns. Together, these findings suggest that TM adoption in this sample is shaped by both structural-economic and perception-based factors, but that the strength and stability of these associations are constrained by sample size and model performance.

### 4.2. Comparison with previous work

The determinants of TM use have been widely discussed internationally, especially in the context of the COVID-19 pandemic,^15,52–54^ which accelerated the adoption of digital health technologies across healthcare systems. Similar patterns have been reported for Germany, particularly in outpatient and primary care settings.^31,38^

This study is consistent with prior work showing the central role of information and education in facilitating TM use among HCPs.^55–57^ Respondents who relied on professional associations or health insurers for guidance were more likely to adopt TM, underlining the importance of trusted institutional actors in providing knowledge and support.^58,59^ Continuous training opportunities and clear, evidence-based communication appear essential to build competence and confidence in TM applications.^60–62^ Professional associations in particular may serve as key multipliers by disseminating best practices, offering targeted education, and creating platforms for exchange. Strengthening these structures through political and institutional supports may accelerate adoption and ensure that TM becomes an established part of routine care.^8,21,63,64^

Perceived patient-related benefits such as improvements in quality of life, adherence, and doctor-patient interaction emerged as relevant factors. This reinforces the notion that physicians’ beliefs about the clinical value of TM may play a central role in adoption decisions. Strengthening the evidence base and communication of these benefits may therefore represent an important lever for increasing uptake.

Reimbursement policies and flexibility have been shown as a critical lever for adoption.^65,66^ However, the lack of adequate and consistent reimbursement still remains a systemic constraint.^20,67^ In our analysis, perceiving inadequate reimbursement as a problem was directly associated with lower TM use, suggesting that economic barriers remain a central obstacle rather than a motivator for engagement. Aligning reimbursement policies with the actual workload and resource requirements of TM is therefore essential to secure its role in routine care.^54,67–70^ Without structural reforms to create sustainable economic incentives, the long-term integration of TM into outpatient care may be limited.

In addition, attitudinal and perception-based factors, particularly physicians’ beliefs about patient benefits, played an important role in the model. This pattern is consistent with broader digital health literature showing that the perceived usefulness and perceived ease of use can balance structural or demographic characteristics in shaping technology adoption.^71–74^ Regional variation, such as negative association observed for Lower Saxony, might suggest uneven uptake across federal states. However, these findings should be interpreted cautiously, as the modest sample size and uneven geographic distribution may introduce sample imbalance, and the observed differences are therefore more likely to reflect contextual variations rather than stable regional determinants.

Overall, our analysis suggests that both structural and economic factors (e.g., technical infrastructure, interoperability, reimbursement, implementation costs) and attitudinal factors (in particular perceptions of patient-related benefits and information needs) shape TM use. This underlines the central role of HCPs in actively promoting TM adoption by offering and endorsing such services.^18,41,75,76^ Engaging strategies should include enhancing digital infrastructure, particularly in underserved regions, and providing targeted training and onboarding to build lasting confidence in TM use.^59,77–80^ TM adoption is also constrained by limited technical infrastructure and the persistent lack of interoperability within and between digital health systems, as highlighted by recent systematic reviews on barriers to TM implementation.^57,81–83^ TM uptake also depends on local, regulatory and reimbursement frameworks in combination with organizational structures at the practice level.^16,75,84–86^ Addressing the needs of groups less inclined to adopt TM, such as older adults or those with limited digital skills or eHealth literacy, and integrating patient and public involvement in designing and implementing TM is essential to ensure equitable uptake and foster perceived and actual usefulness and ease of use.^87–90^

### 4.3. Strengths and limitations

This study has several strengths. The dataset’s strengths lie in its national scope, the inclusion of physicians from multiple specialties, and the timely capture of perspectives during a critical phase of TM implementation. The use of BMA allowed for a principled treatment of model uncertainty, avoiding reliance on a single selected model and providing probabilistic measures of variable inclusion. The integration of a repeated nested CV framework for the ML component strengthened internal validity by reducing optimism bias. In addition, the combination of explanatory and exploratory approaches offered complementary perspectives on TM adoption.

However, several limitations must be acknowledged. The cross-sectional design precludes causal inferences, and the study estimates associations with existing TM use rather than future adoption. Reverse causation cannot be ruled out, and some variables may reflect current practices and attitudes rather than antecedent factors. The absence of external validation limits generalizability. In addition, due to the dissemination of the survey via professional associations, the total number of physicians who received the invitation is unknown, and a response rate could not be calculated, which further limits the assessment of representativeness. At the same time, this distribution strategy enabled broad dissemination across relevant physician groups and facilitated access to a large and diverse professional network. The sample was not evenly distributed across specialties, with cardiologists overrepresented, and the predominance of male respondents may constrain generalizability. Given that the sample is predominantly composed of cardiologists, the findings primarily reflect TM adoption patterns within cardiology-focused outpatient care and may not fully generalize to other medical specialties. Regional effects should also be interpreted with great caution given the uneven geographic distribution. Moreover, as TM infrastructure and regulatory frameworks are rapidly evolving, the findings reflect the situation in Germany in 2023/2024 and may not be fully transferable to future contexts. Lastly, the web-based design may have introduced selection bias, with digital-savvy or TM-interested physicians more likely to participate, and the study did not account for patient-side factors or organizational characteristics at the practice level, both of which are likely to shape TM adoption.

The predictive performance of the XGBoost model was limited, with AUROC values close to chance and confidence intervals crossing 0.50. SHAP-based interpretations should therefore be considered exploratory rather than indicators of predictive importance. The sample size relative to the number of candidate predictors constrains both BMA and ML analyses. In the ML framework, this results in high variance and unstable performance estimates. In the BMA framework, the large model space relative to sample size increases the risk of signal dilution and reduces PIP stability. The use of LASSO prior to BMA further conditions the analysis on a pre-selected subset of variables, which means that the reported PIPs do not reflect uncertainty across the full candidate model space. This limits the theoretical scope of BMA and should be considered when interpreting the results. In addition, the selection of covariates was not guided by a formal theoretical framework, but by the structure of the original survey, which limits mechanistic interpretation. The heuristic approach used to infer SHAP directionality introduces additional uncertainty and should be understood as a pragmatic tool rather than a formal inferential procedure. Nevertheless, comparison with SHAP dependence plots suggests it yields more accurate directional assignments than the conventional approach based on the sign of the mean SHAP value.

Further limitations include the use of single imputation with MissForest, which does not account for imputation uncertainty and may underestimate variability. Multiple imputation would have been preferable. In addition, the TM use variable was constructed from multiple survey items due to the absence of a single direct indicator. Collapsing these heterogeneous modalities into a single binary variable may introduce misclassification and mask differences in use patterns.

### 4.4. Implications

This study has several implications, though these should be interpreted in light of its exploratory nature and the modest predictive performance of the BMA and ML models. Reimbursement schemes, infrastructural investments, and regulatory clarity emerge as candidate levers warranting further investigation in relation to TM adoption. Sustainable financing models that adequately account for physicians’ workload and resource requirements may be necessary to transition TM from an optional complement into an integrated component of routine outpatient care.

Professional associations and insurers appear to serve as key knowledge brokers in this context. By providing clear guidance, evidence-based recommendations, and platforms for best-practice exchange, they may directly influence adoption behavior. Supporting these actors with adequate institutional resources and policy support could help accelerate the diffusion and standardization of TM practices.

For physicians, structured training opportunities that develop both technical competence and confidence in the clinical value of TM for patients may be particularly relevant. Embedding TM into undergraduate medical curricula and continuing professional development programs will likely be crucial for sustained, long-term adoption.

The modest predictive performance of the ML model further suggests that targeted interventions should draw primarily on robust explanatory candidate associations rather than demographic profiling. This underscores the importance of addressing systemic and informational barriers rather than making assumptions based on individual physician characteristics.

Finally, tailored strategies addressing local infrastructural constraints, information gaps, and organizational conditions are likely to be more effective than those targeting demographic subgroups alone. Relevant areas include improving digital literacy, reducing system fragmentation, and aligning financial incentives with diverse practice contexts.

## 5. Conclusion

This study presents a secondary analysis of physician survey data, applying both BMA and ML (XGBoost with SHAP) to identify explanatory candidate associations and exploratory patterns of TM use in the German outpatient sector. Findings suggest that structural–economic factors and attitudinal perceptions, particularly those related to patient-perceived benefits, are more consistently associated with TM adoption than demographic characteristics. Professional associations emerged as potentially influential intermediaries, while physician perceptions of patient-related benefits, including health literacy, quality of life, and trust, showed consistent associations with TM use across both analytical approaches. Given the exploratory nature of these findings, causal interpretation is not warranted; rather, they point to directions for future confirmatory research. Achieving broader integration of TM into routine outpatient care, will likely require coordinated efforts encompassing stable reimbursement, interoperable infrastructure, tailored medical education, and strengthened informational and organizational support, with a focus on addressing systemic and contextual disparities rather than demographic subgroups. Building physician confidence in the clinical value of TM through evidence-based communication, structured and continuous training, and institutional guidance will be essential to ensure sustainable and equitable adoption.

## Data Availability

The study protocol and dataset are available from the corresponding author upon reasonable request. The code used in this study is publicly available at: https://doi.org/10.5281/zenodo.17047856 and https://github.com/PetitPascal/R-scripts.^91^
